# Online Pornography Consumption, Risky Behaviors, and Sexist Attitudes in Adolescence: A Cross-Sectional Survey Study

**DOI:** 10.1007/s10508-025-03217-z

**Published:** 2025-08-20

**Authors:** Sandra Feijóo, Vanessa Portela, Antonio Rial

**Affiliations:** 1 Department of Social Psychology, Basic and Methodology, Universidade de Santiago de Compostela, Santiago de Compostela, Spain; 2 Departamento de Ciencias de la Salud, Facultad de Ciencias de la Salud, Universidad de Burgos, Paseo de los Comendadores, s/n, 09001 Burgos, Spain

**Keywords:** Pornography, Sexual risky behavior, Sexist attitudes, Adolescence, Gender

## Abstract

The present study aimed to analyze the consumption of pornography among adolescents, examine its relationship with sexist attitudes, and explore how its consumption relates to participation in other risky behaviors. The final sample consisted of 664 Galician (Spain) students aged 12–17 years (M = 14.55; SD = 1.70) who completed a survey including ad hoc items about online and offline risky behavior and the Spanish version of the Social Roles Questionnaire. Almost half of the sample (48.8%) had consumed pornography at least once in their lifetime, with 21.7% having done so in the prior month. These rates were significantly higher among males and older students. Those who had used pornography had a significantly higher mean score on the subscale of traditional sexist attitudes and higher engagement in several other risky behaviors. Furthermore, logistic regression analysis showed which of these variables best predicted pornography consumption for females and for males. These results indicate the need for a comprehensive or holistic preventive approach to risky sexual behavior and early sexual education and porn literacy, which leads adolescents to reflect on how pornography may impact their attitudes and relationships.

**Supplementary Information:**

The online version contains supplementary material available at 10.1007/s10508-025-03217-z.

## Introduction

The pervasive use of the Internet among adolescents has amplified their exposure to potentially harmful content, including pornography. Exposure to pornography in adolescence coincides with critical developmental stages, influencing sexual attitudes, risk behaviors, and gendered perceptions (Hald et al., 2013; Montiel et al., 2010; Osorio et al., 2012; Sánchez-González et al., 2021; UNICEF, 2017). Despite the growing body of literature on these issues, comprehensive studies investigating the interconnections between pornography consumption, risky sexual behavior, and sexist attitudes among adolescents remain scarce (Vila-Cortavitarte et al., 2022).

Based on social learning theory (Bandura, 1986), pornographic content may constitute role models and contribute to the development of “sexual scripts,” influencing sexual attitudes and risk engagement (Hald et al., 2013; Wright, 2011; Wright et al., 2023). In the context of mass media effects, the adoption of new sexual scripts from pornography is influenced by multiple moderating and mediating factors that shape the relationship between exposure to pornographic models and potential behavioral outcomes over time (Valkenburg & Peter, 2013). Although Braithwaite et al. (2015) highlighted that the effects of pornography may not be uniformly negative, there are several studies warning about the potential outcomes of its consumption (Bridges et al., 2016; Gallego & Fernández-González, 2019; Montiel et al., 2014; Peter & Valkenburg, 2016; Ybarra et al., 2011). This concern is heightened considering the young age of many of the consumers, the frequency of consumption, or the content represented in pornographic videos. The average age of first access to pornography in Spain was found to be 14.84 years (Ballester & Orte, 2019), although the age drops to 13.19 years if only males are considered (Gallego & Fernández-González, 2019). According to data from the EU Kids Online survey (Smahel et al., 2020), 47% of Spanish males and 34% of females aged between 9 and 16 years had seen sexual images in the prior year. In addition, there appears to be a gender bias in the depicted imagery, since the target was a female in 97% of the cases where physical or verbal assaults were shown in pornography, and their response to aggression was either neutral or positive and rarely negative (Fritz et al., 2020). Consequently, research on the potential effects of pornography covers multiple areas, such as addiction, sexual dysfunction, risky sexual behavior, sexual aggression, and increased gender inequality (Sánchez-González et al., 2021).

In a longitudinal study, Brown et al. (2006) found that exposure to sexual content in the media accelerates adolescent sexual activity, increasing the risk of early sexual intercourse. The more recent meta-analysis of Coyne et al. (2019) obtained similar results. Sexual content in the media influences both sexual attitudes and behavior, as well as acting as a predictor of age of sexual initiation, overall sexual experience, and engagement in risky sexual behavior (e.g., having multiple sexual partners, one-night stands, and no contraception; Coyne et al., 2019). Moreover, multiple risk behaviors tend to converge, giving rise to poly-victimization (Montiel et al., 2014).

Regarding the relationship between pornography consumption and risky behaviors, research has identified several associations. In terms of offline sexual risky behaviors, pornography consumption has been linked to regretting the first sexual experience (Osorio et al., 2012). Using pornography as a source of sexual information has also been linked to increased sexual risk engagement (Rosengard et al., 2012; Wright et al., 2018). Frequent consumption of pornography has been associated with both perpetrating and experiencing sexual assault (Peter & Valkenburg, 2016), engaging in more impersonal sexual acts (e.g., casual, group, or paid sex; Tokunaga et al., 2019), and having more permissive sexual attitudes, multiple partners, or unprotected sex (Harkness et al., 2015; Owens et al., 2012). This last point is the most studied, as pornographic scripts rarely depict condom use (Bridges et al., 2010; Wright et al., 2019). In terms of online risk behaviors, pornography consumption has been associated with sexting (Clancy et al., 2021; Molla et al., 2020; Symons et al., 2018; Van Ouytsel et al., 2014) and contacting strangers online for sexual purposes (Sanjuán, 2020). Sexting, defined as sending, receiving, or forwarding erotic or explicit messages, images, or pictures via electronic devices (Klettke et al., 2014), includes both active (sharing self-generated content) and passive sexting (receiving content; Madigan et al., 2018; Peris & Maganto, 2018). While Montiel et al. (2016) estimated the prevalence of online sexual victimization among Spanish adolescents, including sexual coercion, grooming, unwanted exposure to sexual content, and pressure to share personal information, they did not examine its relationship with pornography consumption.

Another frequently analyzed topic is the relationship between pornography consumption and sexist attitudes, with studies reporting mixed results (Miller et al., 2020). For example, longitudinal analysis have found a relationship between pornography consumption and less progressive gender role attitudes in both males and females (Brown & L’Engle, 2009), a higher perception of women as sexual objects (Peter & Valkenburg, 2009), or an increase in intimate partner violence (IPV) when the male partner in the context of a heterosexual relationship frequently consumed pornography (Jongsma & Timmons Fritz, 2021). Peter and Valkenburg (2016) also found a relationship between sexually explicit internet material consumption and having stronger gender-stereotypical sexual beliefs. Furthermore, Skorska et al. (2018) found that exposure to degrading pornography in an experimental context generated the strongest hostile sexist beliefs and objectification of the actress in the clip. In contrast, other studies have found no relationship between pornography consumption and sexism (Speed et al., 2021) or have found that experimental pornography consumption was only associated with sexism in male participants who scored low on agreeableness (Hald et al., 2013; Miller et al., 2020) or who attributed a high level of perceived realism to pornography (Miller et al., 2020). Inconsistencies between studies may be due to the need to account for multiple moderating variables, as researchers influence their findings by choosing to control or not some covariates (Speed et al., 2021). Gender has also been found to be a moderating factor depending on the variable explored (Ballester & Orte, 2019; Bridges et al., 2016; Gallego & Fernández-González, 2019; Jongsma & Timmons Fritz, 2021), indicating the need for specific analyses by gender.

While prior research has extensively examined the relationships between pornography consumption, sexual risk behaviors, and sexist attitudes independently, there is a lack of integrative studies that address all these variables simultaneously in the same sample, especially with a focus on a young age and gendered differences. Identifying gender-specific predictors of pornography consumption is critical for tailoring effective interventions. Therefore, the present study had the following objectives:

1. Examine age and gender differences in pornography consumption among adolescents.
2. Investigate the relationship between pornography consumption and sexist attitudes.
3. Explore the association between pornography consumption and both online and offline sexual risk behaviors.
4. Identify which variables best predict pornography consumption separately for females and for males.

## Method

### Participants

The sample was selected by convenience, with the collaboration of four secondary schools in the province of Ourense (Galicia, Spain) willing to participate in the study. The initial sample consisted of 754 participants who completed self-administered questionnaires, of which 25 were eliminated for having too many missing values and/or inconsistent response patterns. In addition, 65 participants were eliminated from the final database for being outside the age range targeted in the present study (i.e., being of legal age to consume pornography in Spain, 18 years old). The final sample were 664 adolescents with ages ranging from 12 to 17 years old (mean = 14.55; standard deviation = 1.70), of whom 53.5% self-identified themselves as female, 44.7% as male, and 0.9% as other genders. Regarding their academic grade, 37.9% were in the first or second year of compulsory secondary education (ESO by its Spanish acronym), 34.7% were in the third or fourth year of ESO, 26.1% were studying post-obligatory secondary education (“Bachillerato”), and 1.3% were undergoing a basic vocational training (“FP” by its Spanish acronym).

### Measures

The research team created a custom questionnaire combining validated scales from prior literature in the field with new items developed for the present study. At the beginning of the questionnaire, a series of sociodemographic items were included, which made it possible to draw up a profile of the participants by age, gender, and grade level.

The first section of the questionnaire consisted of items developed by the research team, relating to the consumption of pornography and engagement in other sexual risky behaviors online: “Have you visited websites with erotic or pornographic content?” (pornography consumption); “Have any of your contacts sent you erotic or sexual messages?” (passive sexting via written messages)”; “Have any of your contacts sent you photos or videos of themselves with erotic or sexual content?” (passive sexting via pictures or videos); “Have you sent erotic or sexual messages?” (active sexting via written messages); “Have you sent photos or videos of yourself with erotic or sexual content? (active sexting via pictures or videos); “Have you contacted strangers through the Internet, chats, social networks or video games?” (actively contacting strangers online); “Have you accepted friendship requests on social networks from someone you did not know at all?” (passive contact with strangers online); “Have you met in person with people you met exclusively through the Internet, chats, social networks or video games?” (meeting online acquaintances in person). These items were based on the latest UNICEF report on technology use by adolescents in Spain (UNICEF Spain, 2021).

The second section also consisted of items developed by the research team and included statements about offline sexual risk behavior based on prior research (e.g., Osorio et al., 2012; Tokunaga et al., 2019; Van Ouytsel et al., 2014): “Have you had sex without a condom?”; “Have you had sex that you later regretted?”; “Have you had sex without your full consent?”; “Have you had group sex?”; “Have you or your partner taken the morning-after pill?” The response options included “Never,” or that it had happened at least once during the “Lifetime,” “Last year,” or “Last month.” We must note that we are using “sexual risk behavior” as an umbrella term, but some of these items could be argued to represent negative outcomes instead of risky active engagement on the adolescent side (i.e., regretting the sexual encounter or having to take the morning-after pill). Due to the age of the sample and anticipated difficulties with schools conducting the survey, we decided to inquire about “sex without full consent” instead of using the term “rape” explicitly. Therefore, we will maintain the terminology used in the survey throughout the paper, as we can only infer but not guarantee that the sample had the same understanding as the researchers.

The third section comprised the Social Roles Questionnaire (SRQ-R) (Baber & Tucker, 2006), which allowed the evaluation of sexist attitudes. It was adapted to the Spanish context by López-Cepero et al. (2013). It consists of 13 items with five Likert-type response options, ranging from 0 (“strongly disagree” to 4 “strongly agree”). Cronbach’s alpha (*α*) of the 13 items was 0.78. This questionnaire has two subscales: the Traditional Sexism subscale (*α* = 0.84) and the Gender Transcendence subscale (*α* = 0.76). The Traditional Sexism subscale assesses the degree to which participants associate certain roles with a particular gender (e.g., “For many important jobs, it is better to choose men instead of women”). The Gender Transcendence subscale assesses egalitarian gender attitudes (e.g., “We should stop thinking about whether people are male or female and focus on other characteristics”).

### Procedure

Due to lockdown restrictions linked to the COVID-19 crisis, the research team could not be present during data collection. Instead, this role was carried out by prevention technicians from the target community of the study. In Spain, prevention technicians are a resource of the national education system, hired by municipalities to address different topics in the classroom, such as addiction or bullying. These technicians were previously trained by the research team to collect data. School principals, children, and their parents were informed and gave their consent to the study. Data collection was carried out during the first semester of 2021 in the usual class groups and classrooms of the schools participating in the study. The completion of the questionnaires was voluntary. Students were informed that they had the option to drop out of the study at any moment, and that their data would be treated confidentially and anonymously.

### Data Analysis

The data were recorded, curated, and analyzed using the statistical package IBM SPSS Statistics 28 (IBM Corp. Released, 2021). Chi-square tests (*χ*^2^) were used to assess the associations between pornography consumption and age group, gender, and online and offline risky sexual behaviors. Independent samples *t* tests were performed to compare sexist attitudes between individuals who had consumed pornography in the past year and those who had not. Finally, two binary logistic regression analyses with the forward stepwise (Wald) method were performed to deepen the analysis of the relationship between pornography consumption and other risky behaviors and sexist attitudes in females and males. Pornography consumption was the dependent variable, and all variables previously detected by the *χ*^2^ analysis as significantly associated with pornography consumption were used as the independent variables.

## Results

The results of the present study showed that almost half of the sample (48.8%) had consumed pornography at least once in their lifetime, with 28.1% having done so during the year prior to data gathering and 21.7% only in the prior month. These rates were significantly higher among males and older students, as shown in Table 1. Detailed data on pornography consumption across age groups separated for females and males are included in the Supplementary Material (see Table 4 in the Supplementary Material).

**Table 1:** Pornography consumption

	Overall	Gender				Age (in years)				
		Male	Female	**χ**^**2**^	CC	12–13	14–15	16–17	**χ**^**2**^	CC
Never	51.2%	34.8%	65.5%	60.91**	.31	72%	49.3%	33.5%	68.17**	.32
Lifetime	48.8%	65.2%	34.5%	60.91**	.31	28%	50.7%	66.5%	68.16**	.32
Last year	28.1%	47.7%	11.9%	102.56**	.40	11.5%	30.8%	41.5%	54.76**	.28
Last month	21.7%	40.1%	6.4%	108.20**	.41	7.8%	24.4%	32.1%	44.90**	.25

CC = contingency coefficient

**p < .001

Regarding the potential relationship between pornography consumption and sexist attitudes, the *t* test showed that those who had used pornography in the prior year had significantly higher means in the subscale of traditional sexist attitudes (M = 17.05 vs 15.46; SD = 5.49 vs 5.34; *t* = -3.42; *p* < 0.001; Cohen’s *d* = 0.29), but in the gender-transcendent one, the differences were not statistically significant (M = 8.67 vs 8.31; SD = 4.37 vs 4.38; *t* = -0.95; *p* = 0.34; Cohen’s *d* = 0.08). Goodman and Kruskal’s gamma (*G*) was used to further determine the association between traditional sexist attitudes and pornography consumption, finding a small but significant positive correlation (*G* = 0.19, *p* < 0.001).

Pornography consumption has also shown to be related to a higher engagement in different risky sexual behaviors, both online and offline, as presented in Table 2. The rates of certain behaviors were two to three times higher among those who had consumed pornography compared to those who had not, reaching up to six times higher in the case of sexting.

**Table 2:** Pornography and involvement in risky sexual behaviors in the prior year

	Pornography consumption			
Online behaviors	No	Yes	*χ*^2^	V
Passive sexting (written messages)	13%	52.1%	112.72**	.41
Passive sexting (pictures or videos)	5.3%	32.3%	85.49**	.36
Active sexting (written messages)	3.3%	26.5%	79.75**	.35
Active sexting (pictures or videos)	2.1%	13.6%	33.17**	.23
Receiving sexts originally from a third party	3.7%	20%	45.49**	.27
Suffering pressures or coercions to sext	4.3%	7.9%	2.80	–
Being threatened with sext dissemination	0.4%	1.6%	1.22	–
Receiving sexual solicitations from adults	5.6%	10%	3.58	–
Actively contacting strangers online	32.1%	55.8%	31.31**	.22
Accepting strangers on social networking sites	30.7%	67%	72.21**	.33
Meeting online acquaintances in person	6.2%	16.5%	16.22**	.16
Offline behaviors	No	Yes	*χ*^2^	V
Not using a condom in a sexual relation	6.1%	18.3%	21.49**	.19
Having regrettable sexual relations	1.1%	7.5%	17.87**	.17
Sexual intercourse without own full consent	0.6%	1.6%	0.46	–
Sexual relations in groups	0.4%	3.2%	6.62*	.11
Having to take the morning-after pill	2.3%	4.9%	2.14	–

*p < 05

**p < .001

To further examine the relationship between pornography consumption and other variables, we conducted binary logistic regression analyses separately for females and males, including significant predictors identified in the *χ*^2^ analysis. The resulting models explained pornography consumption through two variables for females (*χ*^2^ = 52.50; *p* < 0.001) and four variables for males (*χ*^2^ = 120.50; *p* < 0.001). As shown in Table 3, the variables included in the final model were the same in both regression models, plus two additional ones for males, and they were all risk factors for pornography consumption; no protective factors could be identified with the variables assessed. The goodness of fit was adequate based on the Hosmer–Lemeshow test for both female (*χ*^2^ = 3.36; *p* = 0.762) and male models (*χ*^2^ = 9.14; *p* = 0.242). The model for females classified correctly 23.7% of pornography consumers, 97.4% of nonconsumers, and explained 28.3% of the variance in the dependent variable (CoxSnell *R*^2^ = 0.142; Nagelkerke *R*^2^ = 0.283), whereas the model for males classified correctly 65.2% of pornography consumers, 84.6% of nonconsumers, and explained 46.6% of the variance in the dependent variable (CoxSnell *R*^2^ = 0.349; Nagelkerke *R*^2^ = 0.466).

**Table 3:** Logistic regression model to predict pornography consumption

Variable	Multiple OR (95% CI)
Female model	
Passive sexting via written messages	2.41 (1.71–3.40)
Passive contact with strangers online	1.74 (1.22–2.50)
Male model	
Passive sexting via written messages	1.63 (1.17–2.27)
Passive contact with strangers online	1.69 (1.32–2.16)
Passive sexting via pictures or videos	3.02 (1.64–5.55)
Not using a condom in a sexual relation	2.28 (1.07–4.90)

OR = Odds ratio; CI = confidence interval

## Discussion

The present study aimed to analyze the consumption of pornography among Galician adolescents, examine the relationship between the consumption of pornography and sexist attitudes, and explore the relationship between the consumption of this type of material and participation in risky behavior, both online and offline.

The results show that almost half of our sample (48.8%) of adolescents aged 12 to 17 years old have consumed porn at least once in their lives, 28.1% in the prior year and two out of ten (21.7%) in the prior month. Males are the main consumers (40.1% vs. 6.4%), and the rates increase as adolescents get older, findings in line with previous literature (Ballester & Orte, 2019; González & Orgaz, 2013; Peter & Valkenburg, 2016; Smahel et al., 2020). However, 28% of children between 12 and 13 years have already accessed porn, opening a debate about early prevention in such a sensitive topic as the sexuality of underage people. There is an absence of quality sexual education and a taboo about discussing the subject with parents, with online content, specifically pornography, being seen as a legitimate information source that influences the sexual socialization of adolescents (Ballester et al., 2022; Hald et al., 2013). This is particularly true for same-sex-attracted adolescents, for whom pornography has traditionally been a source of education when there is no other type of information available (Kubicek et al., 2010). On the other hand, access to pornography in Spain is legally restricted to people over 18 years old (Organic Law 8/2021 (2021), 4 June of Comprehensive Protection from Violence against Children and Adolescents), but all participants of the study who reported consuming pornography were under that age, reflecting the ineffectiveness of such legal prohibition on its own. Sexual education or “pornography/porn literacy” is needed instead in line with the increasingly earlier age of access (Biota et al., 2022), so that when young people start to consume pornography, they can be critical of the content they are watching and reflect on how such content impacts their own attitudes and sexual relationships (Ballester et al., 2022; Rothman et al., 2020). Pornography literacy has also proven useful in decreasing adolescents’ perception that watching porn is a good way to learn about sexual relationships (see Rothman et al., 2018).

Regarding the second objective, the results showed a significant relationship between pornography consumption and a higher score on the traditional gender attitudes subscale, with adolescents who consumed pornography in the prior year being those who assigned certain roles to the genders to a greater extent (17.05 vs. 15.46). Brown and Engle (2009) had already found a relationship between pornography consumption and less progressive gender role attitudes, while Peter and Valkenburg (2016) reported a relationship between consuming pornographic content and having stronger gender-stereotypical sexual beliefs. Mainstream pornography portrays actors in certain roles, showing power relations based on the supremacy of white, Anglo-Saxon, heterosexual, and male over other identities (Pedraz Poza & Ares, 2011), as evidenced by the apparent consent to all their sexual requests (Miller & McBain, 2022), unequal power dynamics, and sexual relations (Carrotte et al., 2020; Klaassen & Peter, 2015). In contrast, no significant differences were observed in the Gender Transcendence attitudes subscale. Both the group that consumes pornography and the group that does not obtain similar scores in the subscale assessed egalitarian gender attitudes. This result could be attributed to several factors. First, the Gender Transcendence attitudes subscale presents a lower reliability as measured by the Cronbach’s alpha (*α* = 0.76) compared to its counterpart (*α* = 0.84), a reason that has led López-Cepero et al. (2013; *α* = 0.47) to exclude this subscale results from their publication. The subscale may not provide an adequate measure of egalitarian attitudes in the Spanish population, or it may not be adequate to measure this construct among people so young (López-Cepero et al., 2013). However, Cronbach’s alpha obtained in the present study was higher than the one obtained by López-Cepero et al. (2013); therefore, we have decided to report our findings with the caveat that they should be interpreted with caution and perhaps the development of new, updated tools for the recording of sexist attitudes in the Spanish population should be considered. Second, this pattern could also be related to a long-standing patriarchal construct that categorizes women into two opposing groups, the “whores” and the “others” (García, 2016), also known as the Madonna–Whore Dichotomy (Bareket et al., 2018). This binary framework may influence how individuals interpret and evaluate gender-related attitudes, potentially leading participants to apply different standards when responding to items measuring traditional sexism (associating women with nurturing and purity, i.e., “Madonnas”) versus gender-transcendent attitudes (which may align with a more sexualized perception, i.e., “Whores”). Consequently, participants in the present study may have evaluated one or the other group of women when responding to items in the sexist and gender-transcendent attitudes subscales.

As for the third objective, analyzing whether there is a relationship between the consumption of pornography and engagement in risky behaviors, we have found a greater participation of adolescents who consume pornography in most of the risky behaviors analyzed. However, no relationship was observed between being pressured to engage in sexting or being blackmailed into disclosing one’s own erotic material, although a relationship was found with having received erotic material from a third party. It could be argued that adolescents themselves may not realize the difference between sexting (self-produced) and pornographic content (retrieved from professional sources). On the other hand, the forwarding of such content may constitute an offense of distribution of child pornography depending on the age of the person portrayed (Article 189, Organic Law 10/1995 (1995), 23 November ), and this may be an aspect not sufficiently addressed with adolescents to raise awareness of the gravity of the issue. Being pressured to sext and suffering sextortion are associated with being in a relationship or having had sexual partners previously, as many cases of sextortion co-occur with teen dating violence (Wolak et al., 2018) or with prior sexting (Agustina & Gómez-Durán, 2016; Alonso, 2017) and not so much with other kinds of risky behaviors, which is perhaps why a relationship with pornography consumption is not observed in this study. Another explanation would be related to a generalized online vulnerability profile characterized by a proneness to engage in online risk behavior but not so much with being victimized themselves, which would still indicate the need for a comprehensive or holistic preventive approach (Beltrán-Catalán et al., 2018; Carbonell & Montiel, 2016; Feijóo et al., 2021).

Risk-taking behavior and the need for holistic prevention go beyond the online setting. In terms of pornography consumption and offline risky sexual behaviors, significant relationships were found with not using condoms, having sex that the adolescent later regrets, and having group sex. All these results are in line with prior research (Ballester et al., 2021; Luder et al., 2011; Osorio et al., 2012; Tokunaga et al., 2019). Although there was a significant relationship with a lack of condom use, there was no relationship with taking the morning-after pill or being forced to have sex without full consent, which may be related to these items being negative outcomes instead of active risky behaviors. These results could be indicative of risky sexual behaviors being learned through pornography, since pornographic scripts rarely include the use of condoms (Bridges et al., 2010; Wright et al., 2019) and group sex has become a more popular search in porn streaming sites (Pornhub Insights, 2022) or point to a more generalized profile of risk exposure or vulnerability, as mentioned above.

The fourth objective of this study was to identify which variables among those with a significant relationship best predicted pornography consumption for females and for males, leveraging the availability of information about engagement in various risk behaviors and gendered attitudes within the same sample. Two binary logistic regression analyses were performed, and the resulting models showed that passive sexting via written messages and passive contact with strangers online were risk factors for both females and males, further supporting the poly-victimization and generalized online vulnerability profile hypothesis (Carbonell & Montiel, 2016; Feijóo et al., 2021; Montiel et al., 2014; Yépez-Tito et al., 2020). It is particularly noteworthy that these behaviors are passive and could be argued to be less concerning than other behaviors with a higher potential to compromise the safety of young people involved, such as meeting their online acquaintances in person. This may relate to an escalation in online risk engagement, similar to legal substance consumption in adolescents leading to the consumption of illegal drugs in what is known as the Gateway Hypothesis (Nkansah-Amankra & Minelli, 2016). However, this is only partly true for males, as the regression model showed two additional variables absent in the female model: passive sexting via pictures or videos and not using a condom during sexual relations. The first could be another step closer to riskier behaviors, while the second relates to the proclivity of males to imitate pornographic sexual scripts and suffer a greater impact on their expectations and stereotypes about sexual relationships (Ballester & Orte, 2019; Bridges et al., 2016).

As a final reflection, we want to highlight that mainstream pornography is a product sold by billionaire companies, not an educational resource, even if young people seem to consider it as such. In this line, it must be noted that searches containing “how to” increased by 244% in Pornhub during the year 2021 (Pornhub Insights, 2022). Pornography itself may not even be the real issue as much as the lack of sources of information for adolescents to turn to, so we must push for the implementation of sexual education based on equality and consent and porn literacy that will help them be critical of such media content (Ballester et al., 2022; Biota et al., 2022; Rothman et al., 2020). At the same time, this echoes a demand made by young people themselves for the inclusion of sexual education in the school curriculum as a subject to be properly taught and assessed, and not merely as a transversal concept, since these are too often overlooked by competent professionals (Leal & Rodríguez, 2020). Children and young people should make the most of their childhoods for as long as possible. However, we must acknowledge that adolescents may want to develop affective relationships and an active sexual life, and they should not have to resort to porn as a replacement mechanism for proper sexual education. Furthermore, it has also been found that the relationship between sexually explicit internet material consumption and sexist views becomes weaker the more adolescents learned about this type of content in their sexual education at school (Vandenbosch & van Oosten, 2017). The report “Rompiendo Moldes” (“Breaking the Mould”) (Rodriguez & Nardini, 2021), for example, proposes a sexual education intervention including content that leads adolescents to question traditional gender roles and the myths of romantic love, cis-heteropatriarchal sexuality, and mainstream pornography, as well as the importance of consent, emotional management, communication, and the caring perspective that all sexual practices should maintain, even casual ones. Finally, gender differences suggest the need for tailored educational modules. In particular, males may require specific interventions to address their higher exposure and susceptibility to pornography-related myths (Ballester & Orte, 2019).

### Limitations and Future Directions

The limitations of this study should be considered when interpreting its findings. First, the sample was drawn from a convenience selection of four schools in a single Galician province. Although we have no reason to expect markedly different trends elsewhere, this limitation affects the generalizability of our results. Additionally, the cross-sectional design hinders causal interpretations between the variables under study, as the relationships between pornography consumption, attitudes, and risky behaviors may be both bidirectional and mutually reinforcing.

Second, while our study focused on victimization and risk exposure, it did not examine the perpetration or harm to others. For instance, we asked whether participants had been pressured to send explicit material but did not ask whether they had pressured others. Future research should address this gap to better understand the interplay between different risk behaviors and inform targeted preventive efforts. Similarly, our focus on risk factors in the regression models means that protective factors, which are key to prevention and intervention, were not explored and warrant further study.

Third, the use of self-reported instruments can impact the interpretation of the constructs. Terms, such as pornography and consent, may not have been uniformly understood by the participants, which could have influenced their responses. Future studies may consider incorporating clearer definitions or pilot testing of the instrument to ensure consistency in comprehension.

Fourth, although we accounted for gender differences by conducting separate binary logistic regression analyses for males and females, age-related effects were not examined in depth. Although we recognize that age can influence the associations under study, our primary focus was on gender differences and the links between pornography consumption, sexist attitudes, and risky sexual behavior. Future research could adopt longitudinal or developmental approaches to explore how these associations evolve over time.

Finally, while our findings align with those of prior research (Ballester et al., 2021; Luder et al., 2011; Osorio et al., 2012; Tokunaga et al., 2019), contradictory evidence exists in the literature. For example, Koletić et al. (2019) found no significant association between pornography consumption and condom use among Croatian adolescents. These discrepancies may reflect cultural and social differences between samples, reflect moderator variables not accounted for, or stem from methodological differences, such as variations in assessment tools, construct definitions, and validity. Future research should aim for greater standardization to improve comparability, and it would be beneficial to examine the specific characteristics of the pornographic material consumed and its content, particularly the portrayal of violence, gender dynamics, and specific sexual practices depicted. Additionally, the role of underlying social constructs (e.g., the Madonna–Whore dichotomy; Bareket et al., 2018; García, 2016) in shaping responses to attitudinal measures should be explored further, as they may influence how participants interpret and report their views on gender and sexuality. Future studies should explore how pornography reinforces heterosexual stereotypes, including male dominance and differential pleasure dynamics, and how these representations shape sexual attitudes and expectations.

## Supplementary Information

Below is the link to the electronic supplementary material.

Supplementary file1 (DOCX 16 KB)
