# Supplementary material for: Online Pornography Consumption, Risky Behaviors, and Sexist Attitudes in Adolescence: A Cross-Sectional Survey Study
Source: Arch Sex Behav. 2025 Aug 20;54(8):3223–33. doi: 10.1007/s10508-025-03217-z (PMC12484347; doi:10.1007/s10508-025-03217-z)
Supplement: Supplementary file 1 — Supplementary file1 (DOCX 16 KB) [file 10508_2025_3217_MOESM1_ESM.docx]

Table 4

Pornography Consumption across Age Groups. Separate data for females and males

| Females | | | | | |
| --- | --- | --- | --- | --- | --- |
|  | Age (in years) | | | | |
|  | 12-13 | 14-15 | 16-17 | **χ²** | CC |
| Never | 81.2% | 65.8% | 50.8% | 29.13** | .27 |
| Lifetime | 13.9% | 20.2% | 33.1% |  |  |
| Last year | 1.6% | 5.3% | 9.7% |  |  |
| Last month | 3.3% | 8.8% | 6.5% |  |  |
| Males | | | | | |
|  | Age (in years) | | | | |
|  | 12-13 | 14-15 | 16-17 | **χ²** | CC |
| Never | 60.4% | 32.7% | 11.3% | 64.20** | .42 |
| Lifetime | 18.7% | 19.2% | 14.4% |  |  |
| Last year | 6.6% | 7.7% | 9.3% |  |  |
| Last month | 14.3% | 40.4% | 64.9% |  |  |

Note: ***p* < .001; CC = Contingency Coefficient
